# Diagnosis of primary amoebic meningoencephalitis by metagenomic next-generation sequencing: A case report

**DOI:** 10.1515/biol-2022-0579

**Published:** 2023-05-23

**Authors:** Xiujuan Che, Zhiyi He, Tao-Hsin Tung, Han Xia, Zhibao Lu

**Affiliations:** Department of Neurology, Neurointensive Care Unit, The Maoming People’s Hospital, Maoming 525000, Guangdong Province, China; Department of Medical Research and Education, Cheng Hsin General Hospital, Taipei, China; Department of Scientific Affairs, Hugobiotech Co., Ltd, Beijing, 100000, China

**Keywords:** *Naegleria fowleri*, primary amoebic meningoencephalitis, metagenomic next-generation sequencing, diagnosis, case report

## Abstract

Primary amoebic meningoencephalitis (PAM) caused by *Naegleria fowleri* is a fatal infection with a mortality rate of more than 95%, despite advances in antimicrobial chemotherapy and supportive care. Initial manifestations of PAM are indistinguishable from bacterial meningitis. Prompt diagnosis and antifungal treatment may help decline the overall mortality. Here we present a case of a 38-year-old man transferred to our hospital due to mild headache, which deteriorated quickly. Severe increased intracranial pressure was found. The cerebrospinal fluid (CSF) was yellowish with significantly increased leukocyte and protein. Smear and culture were negative. The patient was first diagnosed with pyogenic meningoencephalitis. However, the symptoms deteriorated. Metagenomic next-generation sequencing (mNGS) of CSF was applied and finally confirmed *N. fowleri* as the protist pathogen within 24 h. However, due to the time cost of sampling and transportation (2 days), the diagnosis came too late, and the patient passed away 1 day before. In summary, mNGS is a rapid and accurate diagnostic method for clinical practices, especially for rare central nervous system infections. It should be used as quickly as possible for acute infections, such as PAM. All aspects of patient interrogation and prompt identification should be paramount to ensure appropriate treatment and decline the overall mortality.

## 1 Background

Primary amoebic meningoencephalitis (PAM) caused by *Naegleria fowleri* is an acute, fulminant, necrotizing, and hemorrhagic meningoencephalitis, characterized by severe headache, stiff neck, fever (38.5–41°C), altered mental status, seizures, and coma [1,2]. However, the absence of specific clinical evidence of PAM may lead to missed diagnosis and lack of timely treatment. As the most severely affected area was the brain stem, increased intracranial pressure and herniation are usually the causes of death. Regardless of the treatment regimen, the mortality rate of PAM remains approximately 98% [1]. Therefore, early diagnosis is very important for the timely intervention and administration of antibiotics in PAM patients. Unfortunately, the diagnosis of PAM is easily overlooked due to its similar manifestations with bacterial meningoencephalitis and the difficulty of culture of the pathogen. Here we describe a rare case of fulminant PAM caused by *N. fowleri*. The patient was first diagnosed with pyogenic meningoencephalitis, while metagenomic next-generation sequencing (mNGS) finally detected the pathogen.

## 2 Case presentation

A 38-year-old male was transferred to our hospital on October 23rd, 2019 due to fever and headache for 2 days and disturbance of consciousness for 1 day. On the day before admission, he visited the local hospital with chief complaint of high fever and persistent dull headache with severe vomiting. The patient had no history of exposure to freshwater. According to the computer tomography (CT) scan of the head and the cerebrospinal fluid (CSF) test results of the local hospital (Table 1), bacterial meningitis was first considered. Penicillin and ceftriaxone sodium were given. However, the condition was worse, and lethargy appeared.

**Table 1:** Investigations of CSF

CSF	Appearance	Pressure (mmH_2_O)	White blood cell (WBC × 10^6^/L)	Protein (mg/L)	Glucose (mmol/L)	Chloride (mmol/L)
Before admission		150	1,920	1228.6	0.11	111
After admission	Cloudy, yellowish	＞400	104,614	1481.6	1.3	114

On admission, he was conscious but disorientated. The Glasgow score (GCS) was 10. There was pronounced head retraction and opisthotonus with obvious neck rigidity and positive Kernig’s signs. The temperature was 38.0°C. Examination of the nervous system showed no localizing signs. The clinical diagnosis was pyogenic meningitis.

**Figure 1:**
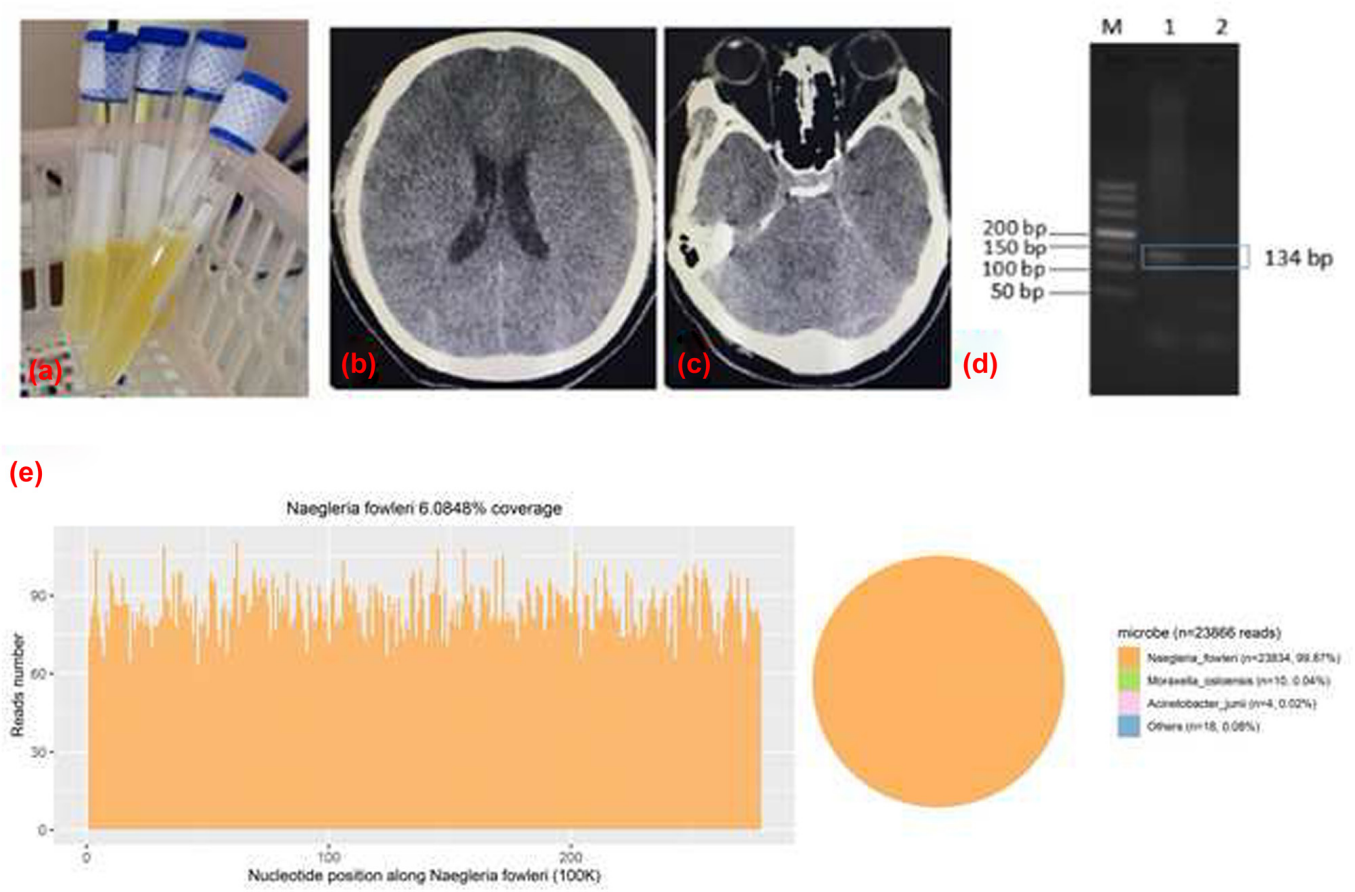
(a) After admission, there showed a cloudy and yellowish CSF. (b and c) Head CT scan showing the decreased density of brain parenchyma, and the narrowed cistern and sulcus, which indicate diffuse swelling of the brain. (d) The physical fragment of *N. fowleri* was 134 bp. The results were consistent with the electrophoretic bands of the patient (Lane 1), and the negative control (Lane 2) had no bands. It is indicated that *Naegleria fowleri* exists in this sample. (e) mNGS results show that a total of 23,834 specific reads of *N. fowleri* were detected in CSF, the coverage was 6.08%.

Three hours after admission, he turned to mild coma (GCS 7) with shortness of breath. Then, mechanical ventilation and sedation were given. Five hours after admission, a lumbar puncture was done. CSF showed significantly elevated white blood cells, and the intracranial pressure was high (Table 1). Sixteen hours after admission, CT revealed diffuse edema of the whole brain (Figure 1b and c). Nineteen hours after admission, he turned to deep coma (GCS 3), and his pupils became dilated.

Two days after admission (October 25th), he remained comatose, with fixed dilated pupils, complete lack of response to stimuli, and spontaneous breathing. The collected CSF sample (2–3 mL) was then sent for PACEseq mNGS (Hugobiotech, Beijing) to detect the pathogen. DNA was extracted and purified from 200 µL CSF supernatant according to the manufacture’s instruction of TIANGEN DNA Mini kit DP316. The DNA library was constructed using QIAseq^TM^ Ultralow Input Library Kit. The concentration and quality of library was checked using Qubit and agarose gel electrophoresis, and the qualified library was then sequenced on Illumina Miniseq platform. A total of 23,834 specific sequence reads of *N. fowleri* were detected by mNGS (Figure 1e). The pathogen was then confirmed by PCR (Figure 1d) using the specific primers F (TCTAGAGATCCAACCAATGG) and R (GTCTTTGTGAAAACATCACC). As a result, the patient was diagnosed with PAM caused by *N. fowleri*. Though mNGS successfully detected the pathogen within 24 h (from October 27th to 28th), too much time had been cost during sampling and transportation (2 days). The patient had passed away on October 26th.


**Informed consent:** Informed consent has been obtained from all individuals included in this study.
**Ethical approval:** The research related to human use has been complied with all the relevant national regulations, institutional policies, and in accordance with the tenets of the Helsinki Declaration, and has been approved by the authors’ institutional review board or equivalent committee.

## 3 Discussion

PAM caused by *N. fowleri* is an acute, fulminant, necrotizing, and hemorrhagic meningoencephalitis [1]. The protist pathogen, *N. fowleri*, is a thermophilic free-living amoeba that can be found in warm lakes and rivers, geothermal springs, naturally hot untreated water supplies, and warm water discharge from industrial plants [1,3,4]. Most patients with PAM experience a history of activities in warm, fresh water which can cause contaminated water entering the nasal cavity [5,6]. The pathogen may then invade the nervous system and cause an infection. PAM cases without water activities were few. It is reported that inhalation of cyst-laden dust is also an important mechanism for PAM, accounting for 6.5% of PAM cases [7], which is extremely concerning. The time of clinical symptoms onset of PAM ranges from 1 to 15 days after exposure [4,8]. Initial manifestations of PAM include fever, severe headache, photophobia, confusion, seizures, and coma, which are indistinguishable from bacterial meningitis [9,10]. In addition, CSF of infected patients is often hazy with increased intracranial pressure, hypoglycorrhachia, elevated protein, and significant pleocytosis (especially neutrophilic predominance), which is also similar to bacterial meningoencephalitis. So, misdiagnosis of PAM is common. However, as brain stem is always the most severely affected area, increased intracranial pressure and herniation are usually the cause of death [11]. So, the timely diagnosis of PAM is needed.

In our case, the patient was presented with milder headache at first, which then aggravated in a very short time. CSF results showed that glucose, protein, and WBC increased significantly, especially WBC. Severe increased intracranial pressure was found in the patient. CSF smear and culture failed to identify the pathogen. Purulent meningoencephalitis was first considered. Despite antibiotics treatment, the disease deteriorated. Considering the poor prognosis, mNGS was applied and the pathogen was detected successfully, indicating PAM. The patient denied a history of swimming outside or any other nasal irrigation, which was different from most reported cases. The patient might be infected by inhaling dust that contained *N. fowleri*, but there is a lack of clear evidence. Further studies are needed to explore the risk factors of PAM.

It is difficult to diagnose PAM using conventional clinical methods. The pathogen is hard to be cultivated and is similar to polymorphs or lymphocytes. PCR-based molecular methods and *in vitro* or *in vivo* animal models are useful methods for the diagnosis [12]. However, there are also limitations. For example, the animal models are always time consuming. PCR is fast with a relatively high sensitivity and specificity, but it needs a prior hypothesis of the target, which is difficult for PAM due to the less typical clinical symptoms.

In recent years, mNGS is increasingly applied for the diagnosis of multiple microbial infectious diseases, especially for rare central nervous system infections, such as leptospirosis and special virus infections [13,14]. It can identify almost all microbial pathogens rapidly and accurately, including bacteria, fungi, mycoplasma, chlamydia, rickettsia, helix, and viruses. Compared with conventional clinical methods, mNGS often has a higher sensitivity and specificity [15]. In this case report, mNGS also successfully detected *N. fowleri* that smear and culture methods failed to identify, indicating its advantage in diagnosing PAM.

Current treatment methods are based on case reports or *in vitro* studies, with limited treatment methods. The therapeutic drugs include amphotericin B, rifampicin, azole (fluconazole), azithromycin, and miltefosine [12]. Dexamethasone, mannitol, and 3% sodium chloride solution or even ventricular drainage may be useful for the severe condition. However, none of these treatments was demonstrated to be effective due to the limited number of survivors. Only about 27% of the cases can be diagnosed before the patient dies. Regardless of the treatment regimen, the mortality rate of PAM remains approximately 98% [1]. The median time from onset to death is 5 days [16].

In our case, CSF smear and culture failed to identify the pathogen. While mNGS rapidly detected the pathogen as *N. fowleri*, which helped in the diagnosis of the patient. mNGS may bring new insight for the rapid diagnosis and help in the timely treatment of PAM patients in the future.
